# Comparing Quality of Life and Psychological Changes in Benign and Malignant Lung Resections

**DOI:** 10.3390/healthcare13010006

**Published:** 2024-12-24

**Authors:** Alin Nicola, Mavrea Adelina, Tamara Mirela Porosnicu, Cristian Oancea, Monica Steluta Marc, Paula Irina Barata

**Affiliations:** 1 Department of Thoracic Surgery, “Victor Babes” University of Medicine and Pharmacy Timisoara, Eftimie Murgu Square 2, 300041 Timisoara, Romania; alin.nicola@umft.ro; 2 Doctoral School, “Victor Babes” University of Medicine and Pharmacy Timisoara, Eftimie Murgu Square 2, 300041 Timisoara, Romania; 3 Department of Internal Medicine I, Cardiology Clinic, “Victor Babes” University of Medicine and Pharmacy Timisoara, Eftimie Murgu Square 2, 300041 Timisoara, Romania; 4 Department of Anesthesia and Intensive Care, “Victor Babes” University of Medicine and Pharmacy Timisoara, Eftimie Murgu Square 2, 300041 Timisoara, Romania; mirela.porosnicu@umft.ro; 5 Center for Research and Innovation in Precision Medicine of Respiratory Diseases, “Victor Babes” University of Medicine and Pharmacy Timisoara, Eftimie Murgu Square 2, Timisoara 300041, Romania; oancea@umft.ro (C.O.); marc.monica@umft.ro (M.S.M.); barata.paula@student.uvvg.ro (P.I.B.); 6 Department of Physiology, Faculty of Medicine, “Vasile Goldis” Western University of Arad, 310025 Arad, Romania

**Keywords:** pulmonary surgical procedures, quality of life, cross-sectional studies, anxiety, depression, lung neoplasms, health status indicators

## Abstract

**Background and Objectives:** Pulmonary resections are critical interventions for treating various lung pathologies, both benign and malignant. Understanding the impact of these surgeries on patients’ Quality of Life (QoL) is essential for optimizing care. This study aims to compare the Health-Related Quality of Life (HRQoL) and psychological well-being in patients who underwent pulmonary resections for benign versus malignant etiologies. **Methods:** A cross-sectional study was conducted involving 117 patients who underwent pulmonary resection between January 2022 and June 2023. Participants were divided into two groups: 51 patients with benign lung conditions and 66 with malignant lung tumors. HRQoL was assessed using the SF-36 and WHOQOL-BREF questionnaires. Anxiety and depression levels were evaluated using the Hospital Anxiety and Depression Scale (HADS) and the Perceived Stress Scale (PSS-10). Patients were assessed pre- and post-intervention. **Results:** Patients with malignant etiologies were older (58.7 vs. 54.2 years) and had lower FEV1% predicted (79.1% vs. 82.5%) compared to the benign group. Malignant patients reported significantly lower scores in physical functioning (68.1 vs. 75.4), role-physical (65.0 vs. 72.3), and general health domains of the SF-36 (62.4 vs. 70.2). WHOQOL-BREF scores indicated a lower overall QoL in the malignant group, particularly in the physical health (65.3 vs. 72.1) and psychological domains (68.0 vs. 74.5). HADS scores revealed higher anxiety (9.1 vs. 7.2) and depression levels (8.5 vs. 6.8) among malignant patients. Correlation analyses showed strong associations between lower QoL scores and higher anxiety and depression levels. **Conclusions:** Pulmonary resections for malignant conditions are associated with a significant decline in HRQoL compared to benign conditions. Patients with malignant etiologies experience higher levels of anxiety and depression, emphasizing that clinicians should integrate specialized mental health services and tailored physical rehabilitation programs for patients undergoing pulmonary resections for malignant lung conditions to address their significantly reduced quality of life and increased psychological distress.

## 1. Introduction

Pulmonary resection surgeries are pivotal in managing various lung diseases, encompassing benign conditions like bronchiectasis, pulmonary hydatidosis, and lung sequestration, as well as malignant tumors such as non-small cell lung carcinoma [1,2]. These surgical interventions aim to excise diseased lung tissue, alleviate symptoms, and, in malignant cases, improve survival rates. However, the impact of these surgeries on patients’ Health-Related Quality of Life (HRQoL) remains a significant concern [3].

The post-operative quality of life can be influenced by several factors, including the underlying disease, extent of resection, surgical approach, and the psychological stress associated with a cancer diagnosis [4]. Patients undergoing surgery for malignant conditions often face additional challenges such as adjuvant therapies, fear of recurrence, and prolonged recovery periods [5]. In contrast, those with benign conditions may experience relief from symptoms and a quicker return to normal activities, potentially leading to better HRQoL outcomes [6].

Previous studies have explored HRQoL in patients after lung resections but have primarily focused on either malignant cases or specific benign conditions [7,8]. There is a paucity of comparative studies assessing HRQoL outcomes between benign and malignant pulmonary resections. Understanding these differences is crucial for tailoring patient care, setting realistic expectations, and improving overall outcomes [9].

Moreover, psychological factors such as anxiety and depression can significantly affect recovery and HRQoL [10]. The stress of a cancer diagnosis can exacerbate these conditions, leading to poorer health outcomes and affecting treatment adherence [11]. Assessing these parameters provides insights into the comprehensive needs of these patients and highlights areas where interventions may improve post-operative well-being [12].

Utilizing the biopsychosocial model, surgical success is assessed not only by clinical factors like complication rates and survival but also by patients’ psychological well-being, including anxiety, depression, and coping strategies. The distinction between benign and malignant conditions introduces additional variables such as the stress of a cancer diagnosis and fear of recurrence, which uniquely affect psychological states. By integrating these elements, the analysis can better contextualize how different surgical outcomes influence the psychological experiences and overall quality of life for each patient group, providing a more comprehensive understanding of their post-operative journeys. Therefore, this study aims to compare HRQoL and psychological well-being in patients undergoing pulmonary resections for benign versus malignant conditions.

## 2. Materials and Methods

### 2.1. Study Design and Participants

A cross-sectional design was chosen to assess these variables simultaneously in both groups pre- and post-operation, providing a clear snapshot of their quality of life and psychological experiences. The study was conducted from January 2022 to January 2024 at two tertiary care hospitals specializing in thoracic surgery and pulmonology. The research protocol was approved by the Institutional Review Board of Victor Babes Hospital (approval number 4459), and all procedures adhered to the Declaration of Helsinki [13]. Written informed consent was obtained from all participants.

A total of 117 patients who underwent pulmonary resection were enrolled in the study. Patients were divided into two groups based on the etiology of their lung condition: benign (*n* = 51) and malignant (*n* = 66). Inclusion criteria were adults aged 18 years or older who had undergone pulmonary resection for a benign or malignant lung condition, were able to provide informed consent, and could complete the study questionnaires independently. Exclusion criteria were precisely defined to ensure the integrity of the study’s results. Individuals with a history of previous lung surgery were excluded to maintain homogeneity regarding surgical impact. Severe comorbidities, which could significantly affect health-related quality of life, were operationalized using standardized diagnostic criteria. Specifically, comorbidities like uncontrolled diabetes were identified using HbA1c levels greater than 8%, and advanced heart failure was defined according to the New York Heart Association (NYHA) Class III or IV criteria. Cognitive impairments were assessed using the Mini-Mental State Examination (MMSE), with a score below 24 serving as a threshold for exclusion due to the potential for hindering effective questionnaire completion. Finally, any individuals who refused to participate were respectfully excluded from the study. Decisions regarding the severity of comorbidities and other exclusion criteria were made by a panel of clinicians specializing in pulmonary medicine and clinical research, ensuring a robust and objective assessment process.

### 2.2. Data Collection and Variables

Demographic data were collected through patient interviews and medical records, including age, gender, marital status, area of residence (urban/rural), occupation, and smoking history (current smoker, former smoker, never smoked). Smoking history was quantified using pack-years. Exposure to respiratory hazards such as asbestos and silica was documented. Data were collected by physicians, nurses, and clinical research coordinators from patient self-reports, electronic health records, and caregiver reports, ensuring a comprehensive approach.

Clinical data encompassed medical history, including comorbidities such as hypertension, diabetes, cardiovascular disease, Chronic Obstructive Pulmonary Disease (COPD), and asthma. Charlson Comorbidity Index (CCI) was calculated to assess the overall comorbidity burden [14]. Surgical history and details were obtained, including indications for surgery, type of resection performed (lobectomy, pneumonectomy, segmentectomy), surgical approach (Video-Assisted Thoracoscopic Surgery [VATS] or thoracotomy), duration of surgery, blood loss, chest drainage details, number and duration of drainage episodes, post-operative analgesia methods, blood transfusions, stays in intensive care, and duration of hospitalizations.

Pre-operative assessments included pulmonary function tests such as Forced Expiratory Volume in one second (FEV1) and Diffusing Capacity of the Lung for Carbon Monoxide (DLCO), performed according to American Thoracic Society guidelines [15]. Cardiovascular evaluations included electrocardiograms (ECGs) and echocardiography to assess left ventricular ejection fraction (EF%). Laboratory tests included complete blood counts and serological tests when indicated.

### 2.3. Assessment Tools

Pulmonary assessments were conducted by a specialized team of physicians, all of whom specialized in pulmonology and thoracic surgery, ensuring the highest levels of reliability and expertise in the evaluations. The health-related quality of life tools, SF-36 and WHOQOL-BREF, were administered in Romanian, with thorough cross-cultural adaptation, validity, and reliability studies ensuring their appropriateness for the Romanian context. These assessments were carried out both pre-operation and post-operation to enable a detailed comparison of health outcomes over time, highlighting significant changes attributable to the surgical interventions.

#### 2.3.1. Health-Related Quality of Life Was Assessed Using Two Standardized Questionnaires

1. SF-36 Health Survey (SF-36): Measures eight domains: physical functioning, role limitations due to physical health, bodily pain, general health perceptions, vitality, social functioning, role limitations due to emotional problems, and mental health. Scores range from 0 to 100, with higher scores indicating better health status [16].
2. WHOQOL-BREF: Assesses four domains: physical health, psychological health, social relationships, and environment. Each domain score ranges from 0 to 100, with higher scores indicating a better quality of life [17].

#### 2.3.2. Psychological Well-Being Was Evaluated Using

1. Hospital Anxiety and Depression Scale (HADS): A 14-item questionnaire with two subscales measuring anxiety and depression. Scores for each subscale range from 0 to 21; higher scores indicate greater levels of anxiety or depression. Scores ≥8 suggest clinically significant symptoms [18].
2. Perceived Stress Scale (PSS-10): A 10-item questionnaire measuring perceived stress over the past month. Total scores range from 0 to 40, with higher scores indicating greater perceived stress [19].

Patients completed the questionnaires during their post-operative follow-up visits at six months after surgery.

### 2.4. Statistical Analysis

Statistical analyses were conducted using SPSS version 26 (IBM Corp., Armonk, NY, USA). Continuous variables were expressed as mean ± standard deviation (SD) and categorical variables as frequencies and percentages. The normality of continuous data was assessed using the Kolmogorov–Smirnov test. A significance level of *p* < 0.05 was set for all statistical tests.

Comparisons between the benign and malignant groups were performed using independent *t*-tests for normally distributed continuous variables and Mann–Whitney U tests for non-normally distributed variables. Chi-square tests or Fisher’s exact tests were used for categorical variables. Effect sizes were calculated using Cohen’s d for continuous variables and Cramér’s V for categorical variables to determine the magnitude of differences between groups [20].

Subgroup analyses were conducted based on age groups (<50 years, 50–60 years, >60 years), gender, smoking status (current/former smoker vs. never smoked), and surgical approach (VATS vs. thoracotomy). Correlations between HRQoL scores and psychological measures were assessed using Pearson’s correlation coefficients. Multiple linear regression analyses were performed to identify independent predictors of HRQoL scores, adjusting for potential confounders such as age, gender, smoking status, and comorbidities.

## 3. Results

### 3.1. Demographics and Clinical Characteristics

Table 1 summarizes the demographic characteristics of the study population. The malignant group was significantly older than the benign group (58.7 ± 7.5 vs. 54.2 ± 8.1 years, *p* = 0.002), suggesting that age is a contributing factor to the development of malignant lung conditions [21]. Gender distribution did not differ significantly between groups, with males comprising 62.7% of the benign group and 68.2% of the malignant group (*p* = 0.504), indicating a higher prevalence of lung diseases requiring resection among males, which aligns with epidemiological data [22].

Marital status and area of residence showed no significant differences between groups. Smoking status, while not statistically significant, revealed that a higher proportion of malignant patients were current or former smokers (47.0%) compared to benign patients (35.3%), supporting the well-established link between smoking and lung cancer risk [23]. Among smokers, the mean pack-years were higher in the malignant group (22.1 ± 8.5) than the benign group (18.5 ± 7.2), although this difference did not reach statistical significance (*p* = 0.087).

Occupational exposure to respiratory hazards was slightly higher in the malignant group (21.2% vs. 15.7%), but the difference was not significant (*p* = 0.394). Charlson Comorbidity Index (CCI) scores indicated that a higher percentage of malignant patients had a CCI score greater than 3 (36.4% vs. 21.6%), suggesting a greater comorbidity burden, although the difference was not statistically significant (*p* = 0.081).

Table 2 details the clinical and surgical characteristics of the patients. Indications for surgery differed significantly between groups (*p* < 0.001). In the benign group, the primary indications were chronic infections (39.2%), bronchiectasis (29.4%), and tuberculosis (19.6%). In contrast, the malignant group predominantly underwent surgery for lung tumors (92.4%). The difference underscores the distinct pathologies necessitating surgical intervention in each group. The types of resection performed did not differ significantly between groups (*p* = 0.645). Lobectomy was the most common procedure in both groups, accounting for 68.6% of benign cases and 60.6% of malignant cases. Pneumonectomy was performed in 19.6% of benign and 22.7% of malignant patients. Segmentectomy was less common but similarly distributed.

Surgical approaches were comparable between groups, with thoracotomy being more common than VATS (64.7% vs. 35.3% in the benign group; 69.7% vs. 30.3% in the malignant group, *p* = 0.564). The choice of surgical approach may reflect the complexity of cases and surgeon preferences. The duration of surgery tended to be longer in the malignant group (3.5 ± 0.9 h) compared to the benign group (3.2 ± 0.8 h), approaching statistical significance (*p* = 0.052). Blood loss was significantly higher in the malignant group (420 ± 180 mL vs. 350 ± 150 mL, *p* = 0.015), possibly due to more extensive resections or tumor vascularity.

Post-operative complications occurred in 30.3% of malignant patients compared to 15.7% of benign patients, a difference that was not statistically significant (*p* = 0.058) but suggests a trend toward higher morbidity in malignant cases. Common complications included prolonged air leak, pneumonia, and atrial fibrillation. Length of hospital stay was significantly longer in the malignant group (11.2 ± 4.1 days) compared to the benign group (9.5 ± 3.2 days, *p* = 0.007), which may reflect the increased complexity and post-operative care needs of malignant patients. These findings highlight the greater surgical burden and resource utilization associated with malignant pulmonary resections.

Table 3 presents the pre-operative pulmonary function tests and assessments. FEV1 (% predicted) was significantly higher in the benign group (82.5 ± 9.0%) compared to the malignant group (79.1 ± 8.5%, *p* = 0.027), indicating better pulmonary function among benign patients. This difference may be due to the underlying pathologies, as malignant conditions can cause more significant lung impairment [24]. DLCO (% predicted) was slightly higher in the benign group (78.2 ± 10.5%) compared to the malignant group (75.0 ± 9.8%), but the difference was not statistically significant (*p* = 0.069). Left ventricular ejection fraction (EF%) assessed by echocardiography showed no significant difference between groups (*p* = 0.071), indicating similar cardiac function pre-operatively.

The prevalence of cardiovascular disease was higher in the malignant group (30.3% vs. 19.6%), though not statistically significant (*p* = 0.179). Respiratory comorbidities such as COPD and asthma were similarly distributed between groups, with no significant differences. The presence of COPD was slightly higher in the malignant group (21.2% vs. 15.7%). Laboratory values revealed a significantly higher hemoglobin level in the benign group (13.5 ± 1.2 g/dL) compared to the malignant group (13.0 ± 1.3 g/dL, *p* = 0.036), which may reflect nutritional status or chronic disease effects in malignant patients [25]. White blood cell counts were comparable between groups.

### 3.2. Survey Analyses

Physical functioning was notably lower in malignant patients (68.1 ± 16.8) compared to benign patients (75.4 ± 15.2, *p* = 0.006), indicating greater limitations in physical activities. Role-physical scores were also significantly lower in the malignant group (65.0 ± 15.9 vs. 72.3 ± 14.7, *p* = 0.008), reflecting more problems with work or daily activities due to physical health. General health perceptions were significantly poorer in malignant patients (62.4 ± 15.8) compared to benign patients (70.2 ± 14.5, *p* = 0.002). This domain assesses personal evaluations of overall health and susceptibility to illness, suggesting that malignant patients perceive their health more negatively. Role-emotional scores were lower in the malignant group (67.8 ± 15.6) than in the benign group (73.5 ± 14.8, *p* = 0.026), indicating more difficulties with work or other activities due to emotional problems. While other domains such as vitality, social functioning, and mental health were lower in malignant patients, the differences were not statistically significant.

**Figure 1:**
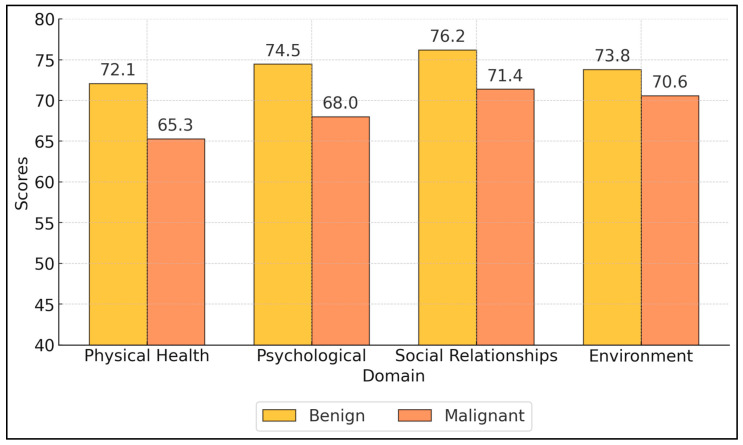
WHOQOL-BREF score comparison between groups.

On the WHOQOL-BREF, the physical health scores were significantly lower in the malignant group (65.3 ± 15.5) compared to the benign group (72.1 ± 14.2, *p* = 0.009), indicating that malignant patients perceive their physical health more negatively. The physical health domain assesses pain, energy, sleep, mobility, and daily activities. The psychological domain was also significantly lower in malignant patients (68.0 ± 14.6) than benign patients (74.5 ± 13.8, *p* = 0.006), reflecting issues with body image, negative feelings, self-esteem, and cognition. These findings suggest that malignant patients experience greater psychological distress. Social relationships scores were lower in the malignant group (71.4 ± 15.7) compared to the benign group (76.2 ± 15.0), but the difference was not statistically significant (*p* = 0.078). The environment domain showed no significant difference between groups, as presented in Figure 1.

**Figure 2:**
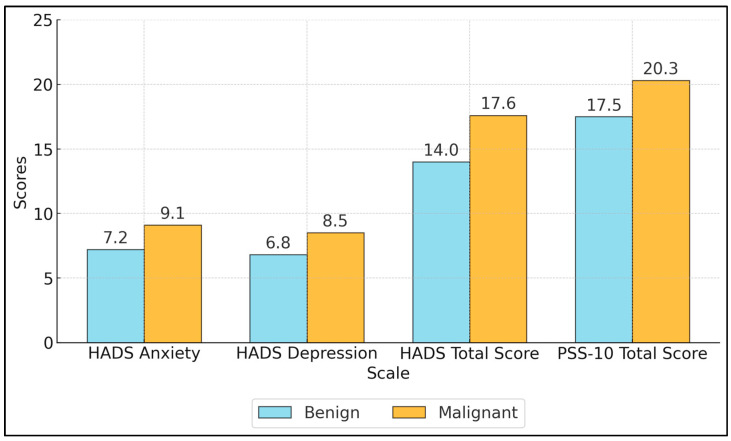
HADS and PSS-10 scores between groups.

The mean anxiety score was 9.1 ± 4.0 in the malignant group versus 7.2 ± 3.5 in the benign group (*p* = 0.002). Similarly, the depression score was higher in the malignant group (8.5 ± 3.7) compared to the benign group (6.8 ± 3.2, *p* = 0.004). The total HADS score, combining anxiety and depression, was significantly higher in malignant patients (17.6 ± 6.5) than benign patients (14.0 ± 5.8, *p* = 0.001), indicating a greater overall psychological burden. Perceived stress levels assessed by the PSS-10 were also higher in malignant patients (20.3 ± 6.0) compared to benign patients (17.5 ± 5.2, *p* = 0.001), as seen in Figure 2 and Table 4. Higher stress levels may be related to the emotional impact of a cancer diagnosis, concerns about prognosis, and the burden of treatment [26].

For the SF-36 measure, both groups showed general improvements across all domains, suggesting an overall enhancement in perceived physical and mental health post-operation. Notably, the Physical Functioning and Bodily Pain scores in both groups increased by 4.5 and 4.2 points, respectively, for benign patients and around 3 points for malignant patients, indicating a significant relief in physical symptoms and constraints. However, the changes in Role-Physical and Mental Health were less pronounced in the malignant group. In the psychological assessments, both groups exhibited decreases in HADS Anxiety and Depression scores, with benign patients showing a slightly greater improvement. The reduction of anxiety and depression by 1.7 points in both groups is statistically significant and clinically relevant, reflecting a successful intervention in terms of emotional and psychological recovery post-surgery. WHOQOL scores also improved, further supporting the enhancement in overall life quality, with Physical Health and Psychological domains improving by about 3 to 4 points. Interestingly, the PSS-10 scores, indicating perceived stress levels, decreased significantly in both groups, with reductions of 2.6 and 2.8 points respectively, as presented in Table 5.

### 3.3. Correlation Analyses

Subgroup analysis based on smoking status revealed that current and former smokers had lower HRQoL scores in both groups, but the differences were more pronounced in the malignant group. Smoking may exacerbate symptoms and impact lung function, leading to poorer quality of life [27].

Patients who underwent VATS reported higher HRQoL scores compared to those who had thoracotomy, regardless of disease etiology. The minimally invasive nature of VATS may result in less post-operative pain, quicker recovery, and better physical functioning [28]. The differences were significant in physical functioning and pain domains. This finding supports the adoption of less invasive surgical techniques when feasible.

Correlation analyses demonstrated strong negative correlations between HRQoL scores and psychological measures. Higher anxiety and depression levels (HADS) and higher perceived stress (PSS-10) were associated with lower physical and mental HRQoL scores (*p* < 0.001). These findings suggest that psychological well-being significantly impacts patients’ perceived quality of life after pulmonary resection. Addressing psychological distress may, therefore, improve HRQoL outcomes (Table 6).

Education level, income, and employment status showed significant positive correlations across most HRQoL domains, highlighting that higher socioeconomic status was associated with better physical and psychological health outcomes. Conversely, negative health behaviors and conditions, such as smoking and higher Charlson Comorbidity Index scores, were significantly correlated with poorer HRQoL across all measured domains. Age also showed a negative correlation with physical aspects of Howl, suggesting a decline in physical functioning and health with advancing age (Table 7).

The multiple regression analysis in Table 1 examines the predictors of SF-36 Physical Functioning among patients undergoing pulmonary resections. The model explains approximately 60.7% of the variance in physical functioning (R^2^ = 0.607, Adjusted R^2^ = 0.584), indicating a strong fit. HADS Total Score (β = −0.412, *p* < 0.001) and PSS-10 Total Score (β = −0.256, *p* = 0.002) are significantly negatively associated with physical functioning, suggesting that higher levels of anxiety, depression, and perceived stress correlate with poorer physical health outcomes. Socioeconomic factors also play a crucial role; Education Level (β = 0.298, *p* = 0.005) and Income (β = 0.347, *p* < 0.001) are positively associated with better physical functioning, highlighting the protective effect of higher socioeconomic status. Additionally, Age (β = −0.198, *p* = 0.010), Smoking Status (β = −0.274, *p* = 0.003), and a CCI Score > 3 (β = −0.325, *p* < 0.001) are negatively related to physical functioning, indicating that older age, smoking, and greater comorbidity burden are linked to reduced physical health), as presented in Table 8.

## 4. Discussion

### 4.1. Literature Findings

This study reveals significant differences in health-related quality of life and psychological well-being between patients undergoing pulmonary resection for benign versus malignant conditions. Patients with malignant etiologies reported lower HRQoL scores across several domains, particularly in physical functioning, general health, and psychological well-being. These findings are consistent with previous research indicating that cancer diagnosis and treatment can adversely affect quality of life [29].

The higher levels of anxiety and depression observed in malignant patients underscore the substantial psychological impact of a cancer diagnosis. The stress associated with malignancy, fear of recurrence, and the burden of treatment may contribute to these heightened psychological symptoms [30]. The strong negative correlations between HRQoL scores and psychological measures suggest that mental health significantly influences patients’ overall well-being. Similar associations have been reported in studies examining the psychosocial aspects of cancer care [31].

Subgroup analyses provided additional insights. The finding that patients undergoing VATS had better HRQoL outcomes aligns with literature supporting the benefits of minimally invasive surgery [32]. The reduced post-operative pain and faster recovery associated with VATS may contribute to improved physical and emotional well-being. Furthermore, the impact of smoking status on HRQoL highlights the importance of smoking cessation interventions as part of comprehensive patient care.

The study’s results emphasize the need for multidisciplinary approaches to patient management. Integrating psychological support services, such as counseling or stress management programs, may alleviate anxiety and depression, potentially improving HRQoL [33]. Pre-operative assessments should include evaluations of psychological status to identify patients at risk of poorer outcomes.

In a similar manner, the study by Li et al. [34] assessed anxiety and depression in patients with incidental pulmonary nodules and found a high incidence of anxiety at 59.3% using the GAD-7 scale. They discovered that social support and a previous history of psychological disease were independent influencing factors for anxiety, with odds ratios (OR) of 2.768 (95% CI: 1.505–5.094) and 5.088 (95% CI: 1.804–14.339), respectively. Additionally, marital status and social support significantly affected depression levels; married patients were less likely to experience depression (OR = 0.375, 95% CI: 0.186–0.754). Similarly, Brunelli et al. [35] evaluated the quality of life before and after major lung resection for lung cancer and observed that the physical composite scale significantly decreased at one month post-operatively (from 51 to 45.1, *p* < 0.0001) but returned to baseline by three months (51 vs. 52.4, *p* = 0.2). They also noted that patients considered at higher risk—such as the elderly, those with poor pulmonary function, or coronary artery disease—had post-operative physical and emotional quality of life scores similar to younger and fitter patients. Both studies highlight the profound impact of lung conditions and surgical interventions on patients’ psychological well-being and quality of life, emphasizing the importance of social support and the need for tailored pre-operative and post-operative care to mitigate negative emotional outcomes.

In their investigation, Sun et al. [36] highlighted the psychological and quality of life challenges faced by lung cancer patients and their family caregivers, proposing a multimedia self-management intervention aimed at preparing them for lung surgery. This approach was driven by evidence suggesting that preparatory education could significantly enhance post-operative recovery and reduce psychological distress. Similarly, the study by Qiu et al. [37] explored the factors influencing surgical decisions among patients with small pulmonary nodules, revealing that anxiety linked to cancer risk and surgical outcomes was a key motivator for patients preferring surgery over the guideline-recommended surveillance. Notably, Qiu et al. [37] found that 234 (69%) of their surveyed patients reported significant anxiety, driven by specific CT scan findings and a familial history of lung cancer, underscoring the psychological burden associated with potential cancer diagnoses and the subsequent decision-making process regarding surgical interventions.

In examining the psychological and physical repercussions of lung cancer surgery, the study by Linares-Moya et al. [38] emphasized the significant impact of pre-operative psychological distress on post-operative outcomes. The research revealed that lung cancer survivors with psychological distress prior to surgery had markedly worse health statuses, functionality, and sleep quality one-year post-resection. Notably, these patients reported higher levels of dyspnea, fatigue, and pain compared to their undistressed counterparts. Similarly, the investigation by Yi-ping Chen et al. [39] explored the immediate post-operative effects of different surgical approaches on lung cancer patients’ psychological status and quality of life. Their findings indicated that, irrespective of the surgical method employed (RATS, VATS, or thoracotomy), patients generally experienced increased depression and a decline in short-term quality of life. Both studies underscore the profound influence of psychological factors on recovery trajectories and quality of life in lung cancer patients, suggesting a critical need for integrated psychological support and tailored perioperative care to optimize patient outcomes in this vulnerable population [40,41,42].

### 4.2. Study Limitations and Future Perspectives

This study has several limitations. The cross-sectional design limits the ability to establish causality between variables. Longitudinal studies are needed to assess changes in HRQoL and psychological well-being over time. The sample size, while adequate for statistical analyses, may not capture all patient variations, and larger multicenter studies could enhance generalizability. Self-reported questionnaires, although validated, may introduce response bias, and patients may underreport or overreport symptoms. Additionally, the study did not extensively analyze factors such as socioeconomic status, educational level, and support systems, which can influence HRQoL and psychological outcomes. We acknowledge that the reliance on self-reported questionnaires presents a limitation, as responses may be influenced by subjective biases or social desirability. Incorporating a more explicit discussion of this limitation earlier in the analysis could provide greater transparency and strengthen the study’s interpretative framework. Future research could benefit from complementing self-reports with objective measures to enhance validity. We also encourage future longitudinal studies to validate findings and investigate QoL changes over time.

## 5. Conclusions

Patients undergoing pulmonary resection for malignant conditions experience a significant decline in health-related quality of life compared to those with benign conditions. The presence of higher anxiety and depression levels in malignant patients highlights the substantial psychological burden associated with cancer diagnosis and treatment. These findings underscore the need for comprehensive care that includes not only surgical management, but also psychological support and interventions aimed at improving mental health. Early identification of at-risk patients and the implementation of targeted support services may mitigate negative impacts on quality of life. Adopting minimally invasive surgical techniques, when appropriate, and promoting smoking cessation may further enhance patient outcomes. Future studies should focus on longitudinal assessments and explore interventions that address both physical and psychological aspects to improve the overall well-being of patients undergoing pulmonary resections.

## Figures and Tables

**Table 1:** Demographic characteristics of patients with benign and malignant lung conditions.

Variable	Benign (*n* = 51)	Malignant (*n* = 66)	*p*-Value
Age (years)	54.2 ± 8.1	58.7 ± 7.5	0.002
Gender (Male), *n* (%)	32 (62.7%)	45 (68.2%)	0.504
Marital Status (Married)	35 (68.6%)	48 (72.7%)	0.621
Residence (Urban), *n* (%)	27 (52.9%)	29 (43.9%)	0.347
Smoking Status, *n* (%)			0.516
Current Smoker	12 (23.5%)	18 (27.3%)	0.219
Former Smoker	6 (11.8%)	13 (19.7%)	0.462
Never Smoked	33 (64.7%)	35 (53.0%)	0.192
Pack-years (if smoker)	18.5 ± 7.2	22.1 ± 8.5	0.087
Occupational Exposure, *n* (%)	8 (15.7%)	14 (21.2%)	0.394
CCI Score > 3, *n* (%)	11 (21.6%)	24 (36.4%)	0.081

CCI—Charlson Comorbidity Index.

**Table 2:** Clinical and surgical characteristics of patients.

Variable	Benign (*n* = 51)	Malignant (*n* = 66)	*p*-Value
Indications for Surgery		6.15 ± 3.28	0.037
Chronic Infection	20 (39.2%)	5 (7.6%)	
Bronchiectasis	15 (29.4%)	0	
Tuberculosis	10 (19.6%)	0	
Lung Tumor	6 (11.8%)	61 (92.4%)	<0.001
Type of Resection			
Lobectomy	35 (68.6%)	40 (60.6%)	
Pneumonectomy	10 (19.6%)	15 (22.7%)	
Segmentectomy	6 (11.8%)	11 (16.7%)	0.645
Surgical Approach			
VATS	18 (35.3%)	20 (30.3%)	
Thoracotomy	33 (64.7%)	46 (69.7%)	0.564
Duration of Surgery (h)	3.2 ± 0.8	3.5 ± 0.9	0.052
Blood Loss (mL)	350 ± 150	420 ± 180	0.015
Post-operative Complications, *n* (%)	8 (15.7%)	20 (30.3%)	0.058
Length of Hospital Stay (days)	9.5 ± 3.2	11.2 ± 4.1	0.007

VATS—Video-Assisted Thoracic Surgery.

**Table 3:** Pulmonary function tests and pre-operative assessments.

Variable	Benign (*n* = 51)	Malignant (*n* = 66)	*p*-Value
FEV1 (% predicted)	82.5 ± 9.0	79.1 ± 8.5	0.027
DLCO (% predicted)	78.2 ± 10.5	75.0 ± 9.8	0.069
EF% (Echocardiography)	57.8 ± 4.2	56.5 ± 4.5	0.071
Cardiovascular Disease, *n* (%)	10 (19.6%)	20 (30.3%)	0.179
Respiratory Comorbidities, *n* (%)			
- COPD	8 (15.7%)	14 (21.2%)	
- Asthma	5 (9.8%)	6 (9.1%)	0.918
Laboratory Values			
- Hemoglobin (g/dL)	13.5 ± 1.2	13.0 ± 1.3	0.036
- WBC Count (×10^9^/L)	7.8 ± 1.5	8.2 ± 1.6	0.104

COPD—Chronic Obstructive Pulmonary Disease; WBC—White Blood Cell.

**Table 4:** SF-36, WHOQOL-BREF, HADS, and PSS score comparison between benign and malignant groups.

Domain	Benign (*n* = 51)	Malignant (*n* = 66)	*p*-Value
SF-36			
Physical Functioning	75.4 ± 15.2	68.1 ± 16.8	0.006
Role-Physical	72.3 ± 14.7	65.0 ± 15.9	0.008
Bodily Pain	68.5 ± 16.3	65.7 ± 17.5	0.348
General Health	70.2 ± 14.5	62.4 ± 15.8	0.002
Vitality	69.8 ± 13.9	66.5 ± 14.7	0.198
Social Functioning	74.6 ± 15.1	70.3 ± 16.2	0.112
Role-Emotional	73.5 ± 14.8	67.8 ± 15.6	0.026
Mental Health	71.9 ± 13.7	68.2 ± 14.9	0.149
WHOQOL-BREF			
Physical Health	72.1 ± 14.2	65.3 ± 15.5	0.009
Psychological	74.5 ± 13.8	68.0 ± 14.6	0.006
Social Relationships	76.2 ± 15.0	71.4 ± 15.7	0.078
Environment	73.8 ± 14.6	70.6 ± 15.2	0.193
HADS			
HADS Anxiety	7.2 ± 3.5	9.1 ± 4.0	0.002
HADS Depression	6.8 ± 3.2	8.5 ± 3.7	0.004
HADS Total Score	14.0 ± 5.8	17.6 ± 6.5	0.001
PSS-10 Total Score	17.5 ± 5.2	20.3 ± 6.0	0.001

SF—Short Form; WHO—World Health Organization; QOL—Quality of Life; HADS—Hospital Anxiety and Depression Scale; PSS—Perceived Stress Scale.

**Table 5:** Within-group pre- and post-operation changes in quality of life and psychological measures.

Measurement	Group	Pre-Operation Mean	Post-Operation Mean	Change	*p*-Value
SF-36 Physical Functioning	Benign	70.9	75.4	4.5	0.012
	Malignant	63.2	68.1	4.9	0.008
SF-36 Role-Physical	Benign	68.4	72.3	3.9	0.03
	Malignant	62.1	65	2.9	0.056
SF-36 Bodily Pain	Benign	64.3	68.5	4.2	0.018
	Malignant	62.9	65.7	2.8	0.07
SF-36 General Health	Benign	66.7	70.2	3.5	0.022
	Malignant	59.8	62.4	2.6	0.089
SF-36 Vitality	Benign	66.3	69.8	3.5	0.045
	Malignant	63.7	66.5	2.8	0.08
SF-36 Social Functioning	Benign	71.2	74.6	3.4	0.035
	Malignant	67.9	70.3	2.4	0.1
SF-36 Role-Emotional	Benign	69.7	73.5	3.8	0.04
	Malignant	65.4	67.8	2.4	0.11
SF-36 Mental Health	Benign	68.4	71.9	3.5	0.03
	Malignant	65.7	68.2	2.5	0.08
WHOQOL Physical Health	Benign	68.1	72.1	4	0.015
	Malignant	62.3	65.3	3	0.05
WHOQOL Psychological	Benign	71.5	74.5	3	0.03
	Malignant	65	68	3	0.045
HADS Anxiety	Benign	8.9	7.2	−1.7	0.008
	Malignant	10.8	9.1	−1.7	0.01
HADS Depression	Benign	8.5	6.8	−1.7	0.006
	Malignant	10.2	8.5	−1.7	0.007
PSS-10 Total Score	Benign	20.1	17.5	−2.6	0.003
	Malignant	23.1	20.3	−2.8	0.001

HADS—Hospital Anxiety and Depression Scale; PSS—Perceived Stress Scale; WHO—World Health Organization; QOL—Quality of Life; SF—Short Form.

**Table 6:** Correlation between HRQoL scores and psychological measures.

Variable	HADS Total Score *	PSS-10 Total Score *	*p*-Value
SF-36 Physical Functioning	−0.62	−0.58	<0.001
SF-36 Mental Health	−0.65	−0.61	<0.001
WHOQOL-BREF Physical Health	−0.59	−0.55	<0.001
WHOQOL-BREF Psychological	−0.69	−0.66	<0.001

WHO—World Health Organization; QOL—Quality of Life; SF—Short Form; PSS—Perceived Stress Scale; HADS—Hospital Anxiety and Depression Scale; *—Correlation Coefficient.

**Table 7:** Correlation analysis of demographic and clinical characteristics.

Variable	SF-36 Physical Functioning	SF-36 Mental Health	WHOQOL Physical Health	WHOQOL Psychological
Education Level	0.47 *	0.43 *	0.52 *	0.46 *
Income	0.53 *	0.37 *	0.59 *	0.51 *
Employment Status	0.39 *	0.31	0.43 *	0.38 *
Age	−0.28 *	−0.17	−0.32 *	−0.26
Gender (Male)	0.12	0.09	0.11	0.07
Marital Status (Married)	0.21	0.19	0.23 *	0.18
Residence (Urban)	0.14	0.1	0.13	0.11
Smoking Status	−0.34 *	−0.29 *	−0.37 *	−0.33 *
Pack-years (if smoker)	−0.41 *	−0.35 *	−0.45 *	−0.39 *
CCI Score > 3	−0.46 *	−0.40 *	−0.48 *	−0.44 *

WHO—World Health Organization; QOL—Quality of Life; SF—Short Form; CCI—Charlson Comorbidity Index; *—Statistically Significant.

**Table 8:** Multiple regression analysis predicting sf-36 physical functioning.

Predictor	β (Standardized Coefficient)	SE	*p*-Value
HADS Total Score	−0.412	0.082	<0.001
PSS-10 Total Score	−0.256	0.074	0.002
Education Level	0.298	0.102	0.005
Income	0.347	0.095	<0.001
Age	−0.198	0.064	0.01
Smoking Status (Smoker)	−0.274	0.088	0.003
CCI Score > 3	−0.325	0.081	<0.001
Constant	48.732		
R^2^	0.607		
Adjusted R^2^	0.584		
F	14.87		<0.001

CCI—Charlson Comorbidity Index; SE—Standard Error; PSS—Perceived Stress Scale; HADS—Hospital Anxiety and Depression Scale.

## Data Availability

The data presented in this study are available on request from the corresponding author.
